# X-ray Scatter Imaging of Hepatocellular Carcinoma in a Mouse Model Using Nanoparticle Contrast Agents

**DOI:** 10.1038/srep15673

**Published:** 2015-10-29

**Authors:** Danielle Rand, Zoltan Derdak, Rolf Carlson, Jack R. Wands, Christoph Rose-Petruck

**Affiliations:** 1 Department of Chemistry, Brown University. 324 Brook Street, Providence, Rhode Island 02912 (USA); 2 The Liver Research Center, Rhode Island Hospital and Warren Alpert Medical School of Brown University. 55 Claverick Street, Providence, Rhode Island 02903 (USA)

## Abstract

Hepatocellular carcinoma (HCC) is one of the most common malignant tumors worldwide and is almost uniformly fatal. Current methods of detection include ultrasound examination and imaging by CT scan or MRI; however, these techniques are problematic in terms of sensitivity and specificity, and the detection of early tumors (<1 cm diameter) has proven elusive. Better, more specific, and more sensitive detection methods are therefore urgently needed. Here we discuss the application of a newly developed x-ray imaging technique called Spatial Frequency Heterodyne Imaging (SFHI) for the early detection of HCC. SFHI uses x-rays scattered by an object to form an image and is more sensitive than conventional absorption-based x-radiography. We show that tissues labeled *in vivo* with gold nanoparticle contrast agents can be detected using SFHI. We also demonstrate that directed targeting and SFHI of HCC tumors in a mouse model is possible through the use of HCC-specific antibodies. The enhanced sensitivity of SFHI relative to currently available techniques enables the x-ray imaging of tumors that are just a few millimeters in diameter and substantially reduces the amount of nanoparticle contrast agent required for intravenous injection relative to absorption-based x-ray imaging.

Hepatocellular carcinoma (HCC) is the fifth most common cancer worldwide and the most common form of liver cancer in adults^1^,^2^,^3^. According to the most recent estimates by the American Cancer Society, over 700,000 new cases of primary liver cancer will develop across the world in 2015, and approximately 600,000 of these cases will result in death^1^. HCC is especially common in developing countries (particularly in sub-Saharan Africa and Southeast Asia), and studies have shown that the incidence of HCC in both the United States and the world is rising^1^,^2^,^3^,^4^.

Unfortunately, HCC is difficult to diagnose in its earliest stages because there are no screening tests available, and the disease usually only becomes symptomatic when the tumor reaches approximately 4.5–8 cm in diameter^1^,^4^. As a result, most patients are diagnosed at advanced stages, and only about 30% of patients present with curative diseases^1^,^2^. Current methods of HCC detection include ultrasound examination and imaging by CT scan or MRI^1^,^5^,^6^,^7^,^8^,^9^,^10^. However, each of these methods has inherent problems, and definitive diagnosis of HCC by these modalities has proven elusive. In particular, the sensitivity of these techniques continues to be problematic, making the detection of early tumors (smaller than approximately one centimeter in diameter) difficult^5^,^6^,^7^,^10^,^11^. Furthermore, low diagnostic specificity has often led to misdiagnosis, resulting in false positive or false negative results that complicate treatment and increase medical costs^2^,^5^,^6^,^7^.

Together, these difficulties in diagnosis contribute to the poor prognosis of HCC, with the American Cancer Society estimating a five-year survival rate of just 15%^1^. However, these numbers are heavily skewed by late stage diagnoses, as cancers detected early in the progression of the disease typically have better outcomes. Patients with small, resectable tumors have a five-year survival rate of over a 50%, and patients with early stage tumors who receive a liver transplant have a five-year survival rate of 60–70%^1^. Improving the prognosis of HCC consequently hinges on being able to detect and diagnose the tumors in their earliest stages^8^.

The development of new techniques for the imaging and early detection of HCC and other cancers is therefore crucial for diagnosis and subsequent treatment. Here we demonstrate that a novel x-ray imaging technique utilizing nanoparticle contrast agents is useful for the noninvasive imaging of liver cancer. The imaging modality described here is a technique called Spatial Frequency Heterodyne Imaging (SFHI) that has been used recently for both biomedical and materials imaging applications^12^,^13^,^14^,^15^,^16^,^17^. SFHI forms an image using x-rays scattered by an object and therefore differs from traditional x-ray imaging, which is based on the differential absorption of x-rays by the sample being studied. Previously published results indicate that SFHI is more sensitive than conventional absorption-based x-ray imaging.

SFHI is similar to other scattering-based x-ray imaging techniques found in the literature that utilize incoherent x-ray sources. Pfieffer *et al.* have shown that conventional x-ray tube sources and absorption gratings such as those used in this study can yield images based on small-angle x-ray scattering that are different from and often complementary to absorption-based x-ray images^18^. Similarly, Wen *et al.* have used the technique to distinguish materials that have identical x-ray absorption properties and to reveal bone structure and density information in rats and pigs^12^,^13^. Others have applied similar types of dark-field or scattering-based x-ray techniques in the biomedical arena, for example investigating the x-ray scattering properties of breast cancer tissue^19^,^20^. However, much of the previous work has focused on x-ray scattering by micron-sized structures^21^. We believe our group has demonstrated the first successful attempt at using sub-100 nm nanoparticles as contrast agents in scattering-based x-ray imaging. Metal nanoparticles such as those used here are very promising as x-ray scatter contrast agents due to their high electron density and large surface area, their small size suitable for intravenous injection, and the ease with which their surfaces can be modified for targeted delivery *in vivo*.

A previous study by our group has shown that SFHI can be used to detect the labeling of HCC tissue with gold nanoparticles (AuNPs) *in vitro*^15^,^16^. We demonstrate here that detection of *in vivo* AuNP-labeled tissues by SFHI is possible in a mouse model. AuNPs have been studied in the past as potential contrast agents for conventional x-ray imaging; they are good candidates because they are nontoxic and have a higher atomic number and x-ray absorption coefficient than typical iodine-based contrast agents^22^,^23^,^24^,^25^. For example, projection x-radiography has been used to detect the biodistribution of small (<2 nm) AuNPs injected intravenously (IV) in a mouse model, and CT imaging has been used to image hepatomas in rat livers labeled with 30 nm AuNPs^22^,^23^. In both of these cases, however, successful imaging required the intravenous injection of large quantities of gold (up to 700 mg Au/kg animal body weight in some cases)^22^. The results described in this manuscript suggest that the enhanced sensitivity of SFHI relative to typical absorption-based x-ray imaging could enable the use of AuNPs as x-ray contrast agents in practical applications by reducing the amount of gold required for visible contrast. In separate studies we have shown that the modality works equally well with many different types of nanoparticles including iron oxide^26^ and gas-filled, virus-like protein shell^27^ nanoparticles. We demonstrate here that HCC tumors can be detected by immuno-labeling the tumors *in vivo* with nanoparticle contrast agents linked to cell specific antibodies, followed by SFHI of the whole animal. The targeted tumors imaged are just a few millimeters in diameter, and are therefore below the detection limits of conventional imaging modalities for HCC.

## Results

### Preparation of nanoparticle contrast agents

For applications *in vivo,* the surface of nanoparticles used as contrast agents must be modified with stable and cell-resistant coatings to help prevent nonspecific uptake of the nanoparticles after injection into the bloodstream. In particular, polyethylene glycol (PEG) is an anti-biofouling coating that has been shown to prevent nonspecific protein adsorption on surfaces, reduce nonspecific cellular uptake of nanoparticles *in vitro*, and increase the circulation times of nanoparticles in the bloodstream^28^,^29^,^30^,^31^. Additionally, when carboxylic acid-functionalized PEG is used as a coating, cell-specific antibodies can be attached to this PEG layer using carbodiimide crosslinking chemistry^32^,^33^,^34^,^35^,^36^. The resulting nanoparticle-antibody conjugates should enable the targeting of specific cancers *in vivo*, delivering contrast agent to the tumors in question while avoiding uptake by other parts of the body^33^,^34^.

For PEGylation of the 10 and 50 nm AuNP contrast agents used here, a heterobifunctional form of polyethylene glycol (HS-PEG-COOH) was utilized; these chains of PEG attach to the surface of AuNPs during mixing due to the affinity of thiols for gold^31^,^36^,^37^,^38^. Addition of PEG to the nanoparticle surface drastically reduces their uptake by cells *in vitro*: when pellets of NIH/3t3 cells of equal size are incubated with equal concentrations of the as-purchased 50 nm Au-cit and the surface-modified 50 nm Au-PEG-COOH, they take up more Au-cit by two orders of magnitude (see Table 1). PEG is therefore successful at imparting on the nanoparticles a quality of “stealth”, which will allow them to circulate through the bloodstream without being easily detected by macrophages^31^,^39^,^40^. This is particularly important for imaging of cancers in the liver, where the high phagocytic activity of macrophages like Kupffer cells may prevent intravenously injected contrast agent from reaching the targeted cancer cells^41^,^42^,^43^,^44^,^45^.

In addition to a nonspecific coating, nanoparticle contrast agents for x-ray imaging of HCC *in vivo* will also require a targeting component that is specific to the cancer being studied. The HCC cell line used here, called the FOCUS cell line, is a good model for the study of directed cancer detection and therapy due to the presence of specific antigens on the FOCUS cell surface that can be recognized by monoclonal antibodies^46^,^47^,^48^,^49^. These cells can therefore be targeted *in vivo* by monoclonal antibodies that bind to the tumor-associated antigens, which should deliver contrast agents to the targeted cells while avoiding uptake by healthy cells. For example, it has been shown that the monoclonal IgG antibody FB50 can be used as a biomarker for HCC due to a strong antibody-antigen interaction between FB50 and aspartyl (asparaginyl)-β-hydroxylase, a protein overexpressed in liver cancer cells^49^.

**Figure 1:**
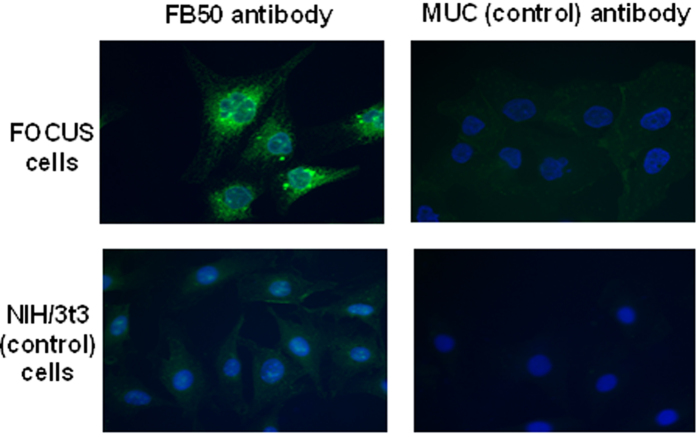
Immunofluorescence study of the labeling of HCC cells with FB50 antibody over control models. Blue staining indicates the positions of the cell nuclei; green staining is a fluorescent tag that indicates the position of antibody in the cell.

The specificity of FB50 for the FOCUS cell line was determined via immunofluorescence. The mouse fibroblast line NIH/3t3 and the antibody for the tropical virus Murutucu (MUC) were used as cell and antibody control, respectively. Immunofluorescence shows enhanced labeling of FOCUS cells with FB50 antibody over control models and indicates that the FB50 antibody targets proteins localized intracellularly (see Fig. 1).

**Figure 2:**
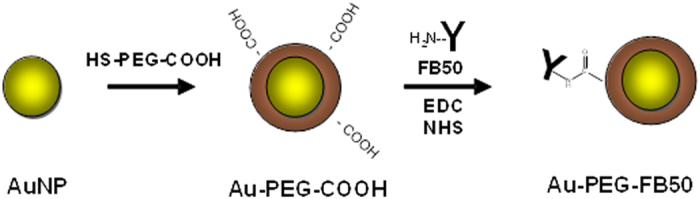
Coating of AuNPs with a layer of functionalized PEG followed by conjugation to the HCC-specific FB50 antibody.

For the development of targeted nanoparticle contrast agents, the FB50 antibody can be conjugated to the surface of Au-PEG-COOH nanoparticle contrast agents using carbodiimide linking chemistry^32^,^33^,^34^. In this process, the carboxylic acid groups on the nanoparticle surface are activated by 1-ethyl-3-(3-dimethylaminopropyl)-carbodiimide (EDC) and N-hydroxysuccinimide (NHS) to form a semi-stable, amine reactive intermediate. Addition of antibody leads to the formation of a bond between an amine side group on the antibody and an activated carboxylic acid group on the nanoparticle surface (see Fig. 2). The amount of antibody added to the activated nanoparticle contrast agents in these studies was enough to yield an antibody: nanoparticle ratio of approximately 1:1 for 10 nm AuNPs and approximately 150:1 for 50 nm AuNPs.

Successful PEGylation of and antibody conjugation to the AuNP surface can be confirmed by measuring the size and zeta potential of the modified nanoparticles. Both measurements were made via dynamic light scattering using a Zetasizer Nano ZS from Malvern Instruments (Malvern, UK) (see Table 2). Both the diameter of the nanoparticles and the zeta potential of the nanoparticles increase with each subsequent step in the preparation of the targeted contrast agent, indicating that both the coating of the AuNPs with PEG and their subsequent conjugation to cell-specific antibodies are successful. Zeta potential of the Au-cit is expected to be negative due to the citrate anions that stabilize the as-purchased AuNPs, but it should increase with the addition of larger, more neutral molecules to the nanoparticle surface.

**Figure 3:**
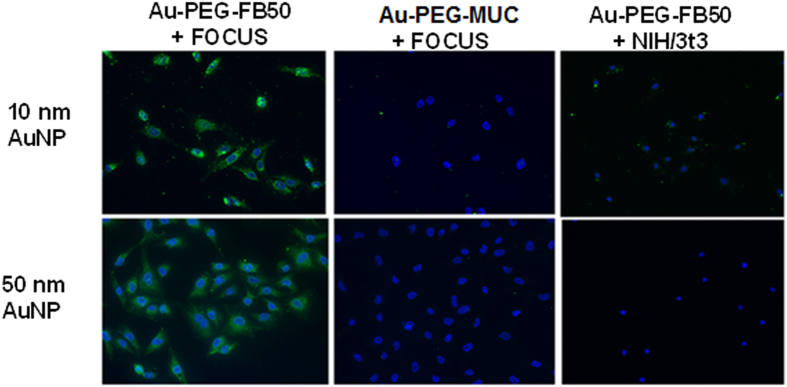
Immunofluorescence study of the labeling of HCC cells with Au-PEG-FB50 conjugates over control models.

Prior to the injection of these targeted contrast agents *in vivo,* it is important to determine whether the FB50 retains its specificity for the FOCUS HCC cell line after conjugation to the nanoparticle. An additional immunofluorescence study indicated that this is the case for both the 10 nm and 50 nm AuNP conjugates; substantial labeling is seen only when the FOCUS cell line is incubated with nanoparticles conjugated to the HCC-specific FB50 antibody (see Fig. 3). Furthermore, this enhanced labeling over control models can be quantified by measuring the integrated optical density of the staining in each fluorescence micrograph, which takes into account both the amount and the brightness of the fluorescent staining of the cells. Measurements of integrated optical density indicate that uptake of Au-PEG-FB50 by the FOCUS cell line is enhanced over control models by an average factor of 11 for 10 nm AuNP conjugates and an average factor of 12 for 50 nm AuNP conjugates (see Table 3).

Overall, the immunofluorescence study shows that the prepared nanoparticle-antibody conjugates have a high specificity for HCC and should be ready for application as contrast agents *in vivo.* The use of targeted nanoparticle suspensions such as those described above should result in an increased cellular uptake of AuNPs at a tumor site, which will in turn lead to enhanced contrast in x-ray scatter images when nanoparticle-labeled tissues are imaged via SFHI.

### Bio-distribution of 50 nm AuNPs

The goal of the first part of our study involving a mouse model was the measurement of the bio-distribution of antibody-conjugated and unconjugated AuNPs. Nude immunodeficient mice were utilized to examine the feasibility of using SFHI for the imaging of biological tissues *in vivo,* and 50 nm AuNPs were selected as contrast agents. This choice was made over the 10 nm AuNP contrast agents due to the higher yield of 50 nm AuNPs after PEGylation and antibody conjugation (reducing materials costs) and the larger x-ray scatter signal provided by 50 nm AuNPs in the x-ray imaging setup described here (reducing the amount of gold required for injection).

The first test of SFHI in an animal model involved three mice and examined the biodistribution of AuNPs in the body 48 h after injection into the bloodstream. To prepare the contrast agent, 50 nm Au-cit were coated with PEG and, in some cases, conjugated to FB50. The Au-PEG-COOH suspension used for injection contained approximately 500 μg of gold, while the Au-PEG-FB50 suspension used for injection contained approximately 300 μg of gold. The lower concentration of gold in the Au-PEG-FB50 suspension is due to the reduced yield of contrast agent after antibody conjugation, which requires an extra synthetic step relative to the PEG coating procedure alone. Of the three mice used in this study, one mouse received an intravenous (IV) injection of Au-PEG-COOH, one mouse received an IV injection of Au-PEG-FB50, and one mouse received an injection of 1 mL sterilized saline as a control.

When nanoparticles larger than approximately 5 nm in diameter are injected into the bloodstream, they are expected to collect mainly in the liver due to the strong phagocytic activity of the liver’s Kupffer cells^45^,^50^,^51^,^52^,^53^,^54^,^55^,^56^,^57^. Due to the presence of these strong phagocytes, IV injections of AuNPs ranging in size from 10 nm to 250 nm have been shown to collect preferentially in the liver in small animal models^45^,^50^,^51^,^52^,^53^,^54^,^55^,^56^,^57^. Nanoparticles can also collect to a smaller extent in other organs including the kidneys, spleen, and lungs^50^,^52^,^53^,^54^,^56^. X-ray imaging in the studies described here was therefore focused on the liver, the kidneys, the spleen, and the lungs.

**Figure 4:**
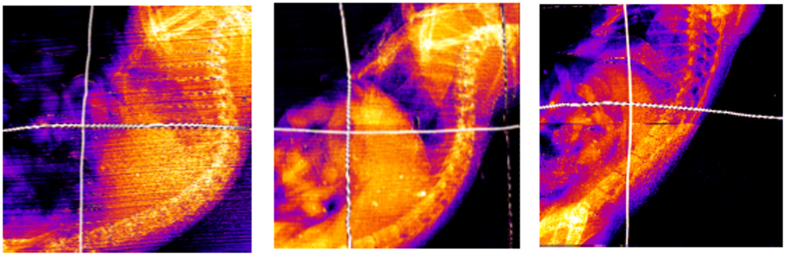
X-ray scatter images of mice 48h after receiving IV injections of saline (left), 50 nm Au-PEG-COOH (middle) and 50 nm Au-PEG-FB50 (right). The crossed wires mark the position of the liver, which should take up significant amounts of AuNPs due to the phagocytic ability of macrophages in the liver.

**Figure 5:**
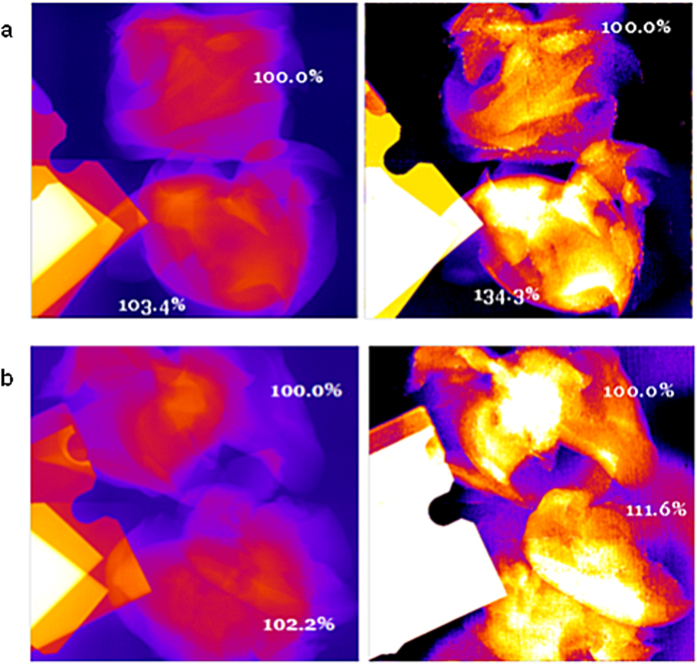
X-ray absorbance images (left) and x-ray scatter images (right) of excised mouse livers. (**a**) The livers were excised from mice that received IV injections of saline (top) and 50 nm Au-PEG-COOH (bottom). (**b**) The livers were excised from mice that received IV injections of saline (top) and 50 nm Au-PEG-FB50 (bottom).

X-ray imaging of the mouse livers *in situ* demonstrated that the presence of gold in these organs can be detected by SFHI. X-ray scatter images of the three mouse livers studied are shown in Fig. 4, where the crossed wires in each image indicate the position of the liver. The average x-ray scatter signal measured in the livers containing AuNPs is six times larger than that measured for the liver devoid of gold. These results were also confirmed by excising each liver and imaging the tissue *ex vivo* (see Fig. 5). In each image, the signal measured for one of the livers containing AuNPs is compared to that measured for the liver devoid of AuNPs. The results indicate that while signal enhancements due to the presence of gold are apparent in both the absorption-based x-ray images and the x-ray scatter images, they are larger in the x-ray scatter images than in the x-ray absorbance images by approximately a factor of 8.

The results also show that liver containing Au-PEG-COOH has a larger x-ray scatter intensity than the liver containing Au-PEG-FB50. This is indicative of the higher gold content in the liver containing Au-PEG, which is most likely due to the higher concentration of gold in the original AuNP suspension that was intravenously injected. It also may be related to the enhanced delivery of the Au-PEG-COOH nanoparticles to the liver relative to the Au-PEG-FB50 nanoparticles, as conjugation of FB50 to the Au-PEG-COOH surface may reduce the anti-biofouling capabilities of the PEG coating and make the nanoparticles more cytophylic.

**Figure 6:**
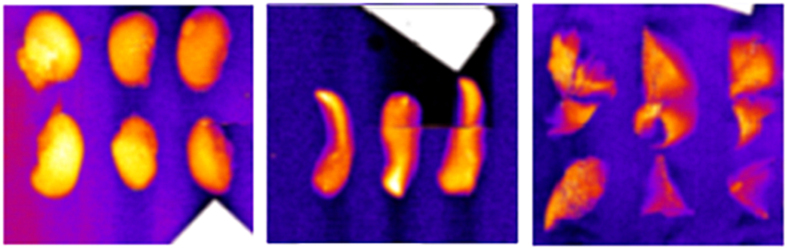
X-ray scatter images of kidneys (left), spleens (middle) and lungs (right) excised from mice. The organs in each image were excised from mice that received IV injections of 50 nm Au-PEG-COOH (left), saline (middle) and 50 nm Au-PEG-FB50 (right).

Kidneys, spleens, and lungs were also excised from the three mice and imaged *ex vivo* by SFHI. X-ray absorbance and x-ray scatter images were taken of each organ; sample x-ray scatter images are shown in Fig. 6. Signal enhancements for the organs containing AuNPs were measured and compared to those measured for the organs without gold in both x-ray absorbance and x-ray scatter images. Those signals are shown in Table 4 along with the signal enhancements measured for the mouse livers imaged *ex vivo*. After x-ray imaging, the gold content in all organs was measured by inductively-coupled plasma atomic emission spectroscopy (ICP-AES) and is shown in Table 4. The liver holds the largest mass of gold on average, taking up more gold than the spleen, kidneys and lungs by a factor of 4, 30, and 100, respectively.

The results in Table 4 show that the only organ showing statistically significant signal enhancement in x-ray images due to the presence of gold is the liver, which correlates with the high gold content in this organ relative to the other three organs studied. Furthermore, the ratio of signal enhancements for the two livers containing gold appears to be correlated to the ratio of the mass of gold contained in each organ. The liver containing Au-PEG-COOH contains an approximately threefold higher mass of gold as the liver containing Au-PEG-FB50, and it also has a measured x-ray scatter signal enhancement that is three times brighter than that measured for the liver containing Au-PEG-FB50. These results suggest that there is a direct correlation between the concentration of gold in nanolabeled biological tissues and their measured x-ray scatter intensities.

**Figure 7:**
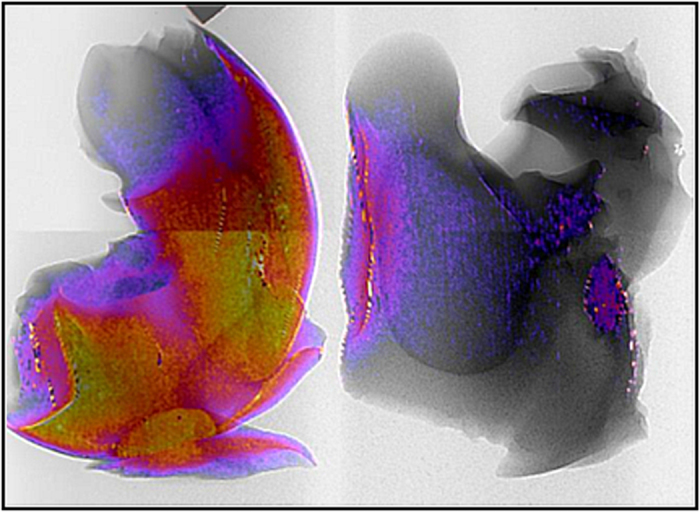
Combined x-ray image of unlabeled (left) and AuNP-labeled (right) mice livers showing SFHI x-ray scatter image (in color) overlaid on top of a conventional x-ray transmittance image (in black and white).

As SFHI produces multiple different types of x-ray images in a single shot, it is also possible to combine these images to obtain information about the sample being studied that may not be apparent with traditional x-ray absorption-based imaging alone. In Fig. 7, an absorption-based x-ray image of a mouse liver labeled with AuNPs is shown in black and white, and an x-ray scatter image of that liver is overlaid on the x-ray absorbance image in color. The resulting image therefore contains features seen in both x-ray absorbance and x-ray scatter images. While x-ray scatter imaging benefits from enhanced sensitivity over traditional x-ray imaging, it also suffers from a loss of resolution relative to absorption-based x-ray imaging due to the image processing required for SFHI. In the dual image shown in Fig. 7, however, the resolution of the absorption-based x-ray image is combined with the sensitivity of the x-ray scatter imaging, which indicates the position of gold labeling in the liver. The combination or comparison of the different x-ray images produced via SFHI may therefore lead to a synergistic effect in which x-ray scatter and x-ray absorbance imaging become complementary rather than competitive, improving image quality and diminishing the disadvantages that limit individual techniques.

### Imaging of nanoparticle-labeled HCC tumors

Having established that the accumulation of AuNPs in biological tissues can be imaged by SFHI, our next project focused on the imaging of HCC tumors that have been grown *in vivo.* A first study involving nonspecific labeling of HCC tumor tissue utilized two nude immunodeficient mice. Three weeks after subcutaneous injection of pellets FOCUS cells into the animals, two tumors had grown in each mouse. Tumor size ranged from 380 mm^3^ to 700 mm^3^, corresponding to tumors that are approximately 0.7 to 0.9 cm in diameter. After the animals were sacrificed, a 50 nm AuNP suspension was injected directly into the tissue of two tumors, while two tumors were left untouched as controls. Both mice were then fixed in formaldehyde for x-ray imaging.

**Figure 8:**
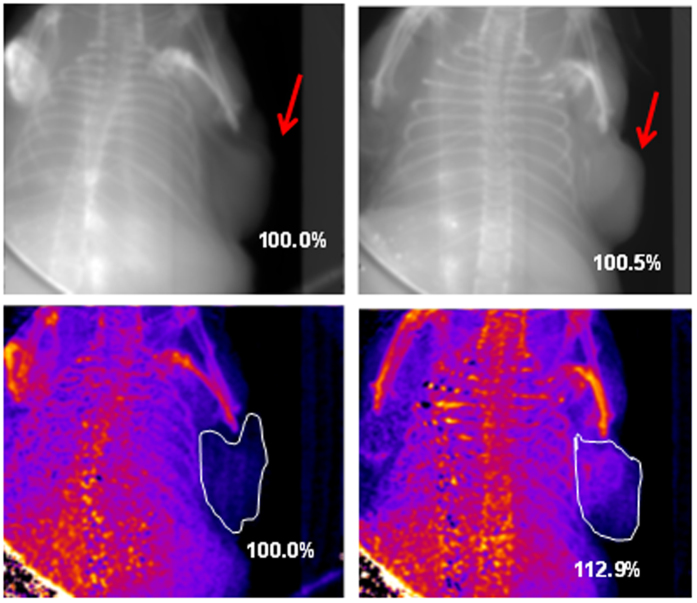
X-ray absorbance (top) and x-ray scatter (bottom) images of HCC tumors *in situ* in mice. The tumor on the right received an injection of 50 nm AuNPs directly into the tumor tissue. The tumor on the left contains no AuNPs.

X-ray absorbance and x-ray scatter images were taken of each tumor *in situ* (see Fig. 8). In each image, the signal in the area of the tumor was measured, and average signal enhancements were calculated for the tumors containing AuNPs relative to the tumor containing no gold. In the x-ray absorbance images, the average signal enhancement for livers containing gold over those devoid of gold is only 0.5%. With x-ray scatter imaging, however, signal enhancements due to the presence of gold improve to 12.9%.

A second x-ray imaging study involved the targeted labeling of HCC tissue *in vivo*. Pellets of FOCUS cells were injected subcutaneously into two mice and allowed to grow for approximately three weeks. Four subcutaneous injections of cells yielded three tumors in the two mice, as one of the xenografts did not grow substantially. Tumor size ranged from 26 mm^3^ to 53 mm^3^, corresponding to tumors that are approximately 3 to 4 mm in diameter.

**Figure 9:**
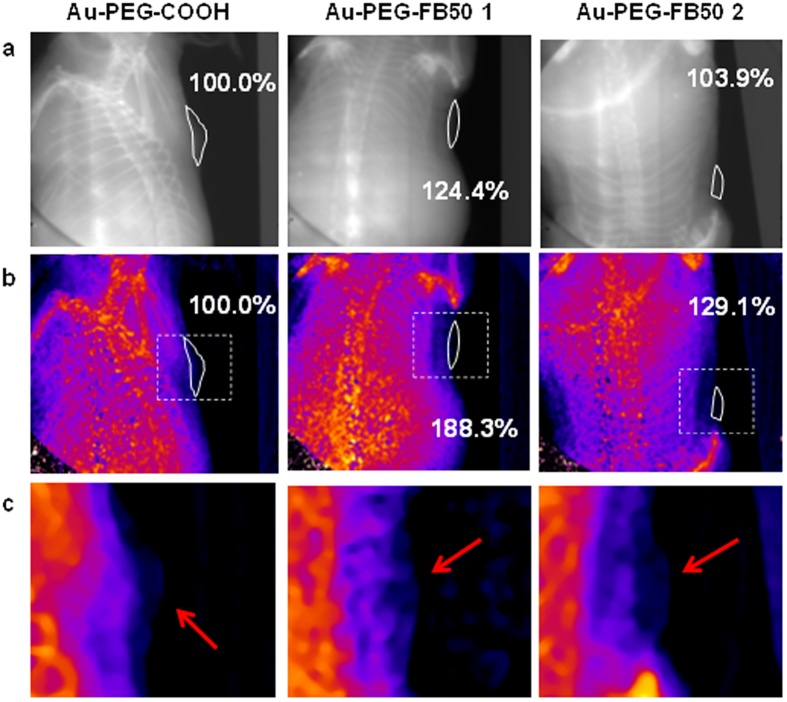
X-ray absorbance (**a**) and x-ray scatter (**b,c**) images of HCC tumors *in situ* in mice. The dotted boxes in (**b**) are magnified in (**c**). All mice received an IV injection of 50 nm AuNPs; images were taken 18 h after IV injection of either Au-PEG-COOH (left) or Au-PEG-FB50 (middle and right). Tumors are identified by white outlines (**a**,**b**) and red arrows (**c**).

For *in vivo* labeling of HCC tissue with contrast agent, suspensions of 50 nm Au-PEG-COOH and 50 nm Au-PEG-FB50 were prepared and injected IV into either mouse. The nanoparticles were allowed to circulate through the bloodstream for 18h before the mice were sacrificed and fixed in formaldehyde for imaging. Absorption-based x-ray images and x-ray scatter images were taken of each tumor (see Fig. 9). In each image, the signal in the area of the tumor was measured, and average signal enhancements were calculated for the tumors containing targeted AuNPs (Au-PEG-FB50) relative to the tumor containing untargeted AuNPs (Au-PEG-COOH). Similar to the results seen in the images of the mouse livers and HCC tumors described above, signal enhancements are apparent in both types of x-ray images but are larger in x-ray scatter images by an average factor of 6. These results indicate that SFHI is more sensitive than traditional absorption-based x-ray imaging to the labeling of HCC tissue with gold nanoparticles *in vivo.* Furthermore, the average x-ray scatter signal for the tumors in the mice that received FB50-targeted AuNPs is 58.7% higher than that measured for the tumor in the mouse that received only PEGylated AuNPs. These results demonstrate that conjugation of FB50 to the nanoparticle contrast agents is successful at enhancing the delivery of AuNPs to the tumor site. It is also important to note that all tumors imaged are labeled by AuNP contrast agents; the control in this study is a tumor that was labeled nonspecifically by untargeted Au-PEG-COOH. We can therefore reasonably expect the signal enhancements to increase when a tumor devoid of gold is used as a control.

To confirm the labeling of these tumors with AuNPs and to quantify the ability of the FB50 antibody to deliver contrast agent to the tumor site, the HCC tumors and the livers were excised from each mouse and tested for gold content (see Table 5). Both tumors in the mouse that received an IV injection of targeted Au-PEG-FB50 have higher concentrations of gold than the tumor in the mouse that received an IV injection of untargeted Au-PEG-COOH, receiving 40% more gold on average. This indicates that conjugation of the FB50 to the AuNPs has a positive effect in delivery gold to the tumor site.

Furthermore, analysis of gold content in the corresponding livers indicates that the liver from the mouse receiving an IV injection of Au-PEG-COOH took up more AuNPs nonspecifically than the tumor excised from the mouse receiving an IV injection of Au-PEG-FB50. This is important, as the early detection of HCC in a mouse or human model will eventually require the imaging of tumors that are found not subcutaneously but growing within the liver. The amount of gold that reaches the tumor relative to the amount that reaches the liver will therefore be very important, as high contrast between the two will be needed for reliable tumor detection. The data here show that both tumors labeled with Au-PEG-FB50 contain a higher concentration of gold than the corresponding liver from that same mouse, while the tumor labeled with Au-PEG-COOH has a lower gold concentration than the corresponding liver.

## Discussion

**Figure 10:**
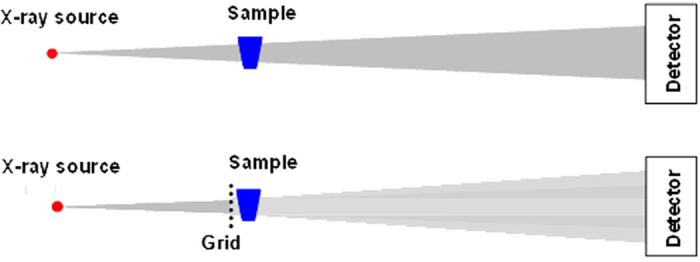
Imaging setup for absorption-based x-ray imaging (top) and SFHI (bottom). Insertion of an absorption grid between x-ray source and sample allows for the detection of x-rays scattered away from the primary beam direction.

Spatial Frequency Heterodyne Imaging (SFHI)^12^,^13^,^14^,^15^,^16^,^17^ is an x-ray imaging technique that is based on a linear arrangement of x-ray source, object, and x-ray detector, much like that of a conventional x-ray imaging apparatus. However, this novel modality rests on a complete paradigm reversal compared to conventional x-ray absorption-based radiology, as it uses x-rays scattered by the object (which are typically rejected in conventional x-radiology) to form an image. The imaging setup therefore differs slightly from that of traditional absorption-based x-ray imaging (see Fig. 10). In SFHI, the deflection of x-rays from the primary beam direction is detected by placing an absorption grid between the object and the x-ray source. While an image taken of the grid alone is very sharp, the introduction of an object leads to x-ray scattering that causes a blurring of the grid image. The extent of this blurring can be measured via numerical Fourier processing of the blurred image, and it corresponds to the amount of x-rays that are scattered by the object. The theory behind the technique and the image processing procedure are discussed elsewhere^15^,^16^,^17^.

Fourier processing of the blurred image yields multiple images that contain different information regarding how the sample being imaged interacts with incident x-rays. Specifically, each single x-ray exposure taken via SFHI produces two types of x-ray scatter images (one corresponding to x-radiation scattered horizontally by the sample and one corresponding to x-radiation scattered vertically by the sample) as well as an image corresponding to x-radiation absorbed by the sample. As such, SFHI enables x-ray scatter imaging while at the same time retaining the ability to provide traditional absorption-based x-ray images. It is also important to note that placement of the absorption grid between the x-ray source and the sample being imaged would reduce radiation exposure to the patient in corresponding biomedical x-ray imaging applications.

SFHI is presented in this manuscript as a method for the early detection of HCC. The data presented here demonstrate that IV injections of AuNP contrast agents can allow for successful x-ray scatter imaging of tissues (and specifically HCC tumors) in a mouse model. Direct targeting of HCC cells *in vivo* using the HCC-specific FB50 antibody seems to improve delivery of gold to the tumor site, and SFHI is sensitive enough to detect small concentrations of gold contrast agent in tumors that are just a few mm in size. Overall, the sensitivity and potential specificity of the novel nanoparticle-based imaging technique proposed here make it a promising method for the early detection and diagnosis of cancers such as HCC. SFHI can detect the labeling of liver cancer tissue with AuNPs both *in vitro* and *in vivo*, even at relatively low concentrations of gold. Additionally, the models of HCC examined here are only 3 to 4 mm in diameter. The results therefore show that SFHI can be used to detect HCC below current detection limits, as ultrasound imaging, CT and MRI often have trouble detecting HCC tumors below about 1 cm in size^5^,^6^,^7^,^10^,^11^.

Furthermore, because the arrangement of source, patient, and detector in SFHI is identical to that used for conventional x-ray imaging, the process to upgrade existing x-ray machines for SFHI service will be straightforward. The modifications made to conventional x-ray machines will not affect their ability to provide absorption-based images and, as such, it will be possible to deliver both x-ray absorbance and x-ray scatter images using a single x-ray apparatus. Tests of SFHI in a clinical setting are currently underway using a GE 100a XRI projection x-ray source at Massachusetts General Hospital. This equipment (typically used for chest radiography) has a spot size of 300 μm and x-ray energies of approximately 70 keV. SFHI of a variety of samples was successful on this machine with exposure times of only 12.5 ms, indicating the potential for application of SFHI *in vivo* in human patients using clinically-available x-ray sources.

Future studies will require a larger population of subjects for SFHI and should involve the targeting and x-ray imaging of tumors grown within the liver for realistic applications in the detection of HCC. In these studies, it will also be important to improve the delivery of contrast agent to the tumor even further. This could be accomplished by reducing the phagocytic activity of Kupffer cells in the liver by injecting phagocytic blockers into the bloodstream immediately prior to the injection of contrast agents; compounds such as dextran sulfate, gadolinium chloride, and even anti-macrophage antibodies and receptor antagonists have been shown to suppress the function of Kupffer cells^58^,^59^,^60^,^61^. It should also be possible to alter the timing of contrast agent injection and subsequent x-ray imaging to find the optimum time point at which specific uptake of nanoparticles at the tumor site is high, but nonspecific uptake of nanoparticles by the liver is low.

As shown here and in previous studies, SFHI is more sensitive than typical absorption-based x-ray imaging. It therefore may have the potential to detect tumors significantly below current detection limits—namely, when they are just a few millimeters in size. The enhanced sensitivity of SFHI should also reduce the amount of contrast agent required for intravenous injection relative to currently available techniques. For example, the mass of gold injected for the x-ray scatter imaging of the HCC tumors described here is one to two orders of magnitude lower than that injected for similar studies using conventional absorption-based x-radiography^22^,^23^.

While SFHI has been applied here for the detection of HCC, it is a versatile technique that is not specific to the imaging of liver cancer. The novel technique described here could be applied to the imaging and diagnosis of a number of different types of cancers as well as other diseases. Furthermore, SFHI is not specific to gold for contrast; recent studies have shown that nanoparticle contrast agents with both very high and very low electron density (such as iron oxide nanoparticles and protein-based nanobubbles) are suitable as contrast agents in x-ray scatter imaging^26^,^27^. It may therefore be possible to utilize nanoparticles with both imaging and therapeutic capabilities for cancer theranostics involving SFHI.

## Methods

### Materials

10 nm and 50 nm gold nanoparticles (AuNPs) stabilized by citrate buffer (referred to as Au-cit) were purchased from British Biocell International (Cardiff, UK). All other chemicals were purchased from Sigma Aldrich (St. Louis, MO).

### Coating of gold nanoparticles with poly(ethylene glycol)

10 and 50 nm AuNP contrast agents were coated with a heterobifunctional form of polyethylene glycol (PEG) to make them more biocompatible. *O-*(3-carboxypropyl)-*O’-*[2-(3-mercaptopropionylamino)ethyl]-polyethylene glycol (HS-PEG-COOH, M_w  _= 3000) was prepared at 100 μM in nanopure water. To 1 mL of Au-cit, either 290 μL (for 10 nm nanoparticles) or 57 μL (for 50 nm nm nm nanoparticles) of this HS-PEG-COOH solution was added, mixed, and left to react overnight at room temperature. The amount of PEG used corresponds to approximately 22 chains of PEG per nm^2^ surface area of gold (about 7000 chains for each 10 nm AuNP, or 173,000 chains for each 50 nm AuNP). Excess PEG was removed by centrifugation, and the coated nanoparticles were resuspended in nanopure water. The resulting PEGylated AuNPs will be referred to as Au-PEG-COOH.

### Conjugation of PEGylated nanoparticles to antibodies

Carbodiimide linking chemistry was utilized for conjugation of the PEGylated AuNPs to antibody targeting agents. 1-ethyl-3-(3-dimethyla-minopropyl)-carbodiimide (EDC) and N-hydroxysuccinimide (NHS) were prepared at 10 mM in nanopure water. To each 1 mL of Au-PEG-COOH, 5 μL of NHS and 5 μL of EDC (for 10 nm AuNPs) or 0.9 μL of NHS and 0.9 μL of EDC (for 50 nm AuNPs) were added and stirred for 1 h at room temperature. After 1 h reaction time, excess EDC and NHS were removed by centrifugation and washing with ultrapure water, and the resulting nanoparticles were resuspended in phosphate buffered saline (PBS). The monoclonal antibody FB50 (specific for Hepatocellular Carcinoma) or the tropical virus antibody Murutucu (MUC, the control antibody) were added to the activated AuNP suspensions and stirred for 1 h at room temperature. The antibody-conjugated AuNPs were stored at 4 °C and are referred to as Au-PEG-FB50 and Au-PEG-MUC, respectively.

### Cell culture

The cancer cell line used is a human Hepatocellular Carcinoma (HCC) cell line called FOCUS, which was obtained from the Wands Lab (Brown University/Rhode Island Hospital, Providence, RI)^62^. Cells were maintained at 37 °C in an atmosphere of 5% CO_2_ in Eagle’s Minimum Essential Medium (EMEM) supplemented with 10% fetal bovine serum, 1% penicillin/streptomycin and 1% L-glutamine. The control cell line used was a mouse fibroblast cell line called NIH/3t3. NIH/3t3 cells were maintained at 37 °C in an atmosphere of 5% CO_2_ in Dulbecco’s Modified Eagle Medium (DMEM) supplemented with 10% fetal bovine serum, 1% penicillin/streptomycin and 1% L-glutamine.

### Cellular uptake of nanoparticles

NIH/3t3 cells were used to determine the effect of surface coating on cellular uptake of nanoparticles. Cells were collected into pellets of 2 × 10^6^ cells and incubated with equal concentrations of 50 nm Au-cit and 50 nm Au-PEG-COOH for 1 h at room temperature. After 1 h incubation time, the cells were washed with PBS to remove any excess AuNPs, and gold content in the cells was measured by inductively-coupled plasma atomic emission spectroscopy (ICP-AES).

### Immunofluorescence

Immunofluorescence studies determined the specificities of the antibodies and antibody-conjugated nanoparticles for HCC and control cell lines. FOCUS and NIH/3t3 cells were plated in 35 mm wells at a density of approximately 2 × 10^5^ cells per well. Cells were fixed with 2% paraformaldehyde and permeabilized by incubation in 0.1% Triton-X 100 in PBS. Cells were incubated with primary antibody (either free FB50 and MUC or AuNP-conjugated FB50 and MUC) for 2 h at room temperature, followed by 2 h incubation with a fluorescein isothiocyanate-labeled secondary antibody used for detection.

### Animal model

Seven nude immunodeficient mice were utilized for labeling of tissues with AuNPs *in vivo* and subsequent x-ray imaging. All experimental protocols involving live animals were approved by the Lifespan Animal Welfare Committee of Rhode Island Hospital in Providence, RI and were carried out in accordance with NIH Guidelines for the Care and Use of Laboratory Animals.

### Biodistribution study

To determine the biodistribution of AuNPs in a mouse model after intravenous (IV) injection, three mice were utilized. Suspensions of 50 nm Au-PEG-COOH and 50 nm Au-PEG-FB50 were prepared at 500 and 300 μg gold/mL in sterilized saline, respectively. One mouse received an IV injection 1 mL Au-PEG-COOH, one mouse received an IV injection of 1 mL of Au-PEG-FB50, and one mouse received an IV injection of 1 mL sterilized saline as a control. All injections were made into the tail vein. Injections were done in two 0.5-mL portions made 24 hours apart. Animals were sacrificed 24 h after the second IV injection (for a total circulation time of 48 h) before being fixed in formaldehyde for x-ray imaging.

### Imaging of HCC tumors

For nonspecific labeling of HCC tumors with AuNPs, two mice were utilized. Tumors were grown from small pellets of FOCUS cells injected subcutaneously in each mouse. For preparation of the xenograft, FOCUS cells were grown to near confluency and collected into pellets of 3 × 10^6^ cells. These pellets were resuspended in serum-free EMEM and injected just under the skin behind the shoulder of each mouse. Each mouse received two subcutaneous injections for a total of four potential HCC tumor growth sites. Tumors were allowed to grow and solidify for three weeks prior to sacrifice of the animals and direct injection of AuNP contrast agents into the tumor tissue. 50 nm AuNPs stabilized by citrate were collected into aliquots of 100 μL volume containing approximately 400 μg gold and were injected into two of the tumors; two tumors received no injection of AuNPs as controls.

For the targeted labeling of HCC tumors with AuNPs, two mice were utilized. Tumors were grown from small pellets of FOCUS cells injected subcutaneously in each mouse. For preparation of the xenograft, FOCUS cells were grown to near confluency and collected into pellets of 3 × 10^6^ cells. These pellets were resuspended in serum-free EMEM and injected just under the skin behind the shoulder of each mouse. Each mouse received two subcutaneous injections for a total of four potential HCC tumor growth sites. Tumors were allowed to grow and solidify for three weeks prior to IV injection of AuNP contrast agents. Suspensions of 50 nm Au-PEG-COOH and 50 nm Au-PEG-FB50 were prepared at 200 μg gold/mL in sterilized saline. One mouse received an IV injection of 0.5 mL Au-PEG-FB50, and one mouse received an IV injection of 0.5 mL Au-PEG-COOH as a control. All injections were made into the tail vein. Animals were sacrificed 18 h after IV injection of the AuNPs before being fixed in formaldehyde for x-ray imaging.

### X-ray Spatial Frequency Heterodyne Imaging (SFHI)

All x-ray measurements made using a microfocus x-ray tube (Trufocus Corp., model TFX-3110EW) with a tungsten anode. The tube was typically operated at an electrical power of 15–20 W, with an anode voltage of 80–100 kV. The high voltage is used to reduce required exposure times; it is also better suited for applications requiring large penetration depths. For all measurements, the distance between the x-ray source and detector was fixed at 1.6 m, while the distance between the x-ray source and the sample being imaged varied; images were typically taken at a magnification of 2 (with the sample placed at 0.8 m) or a magnification of 4 (with the sample placed 0.4 m). The absorption grids used are nickel wire meshes purchased from Structure Probe, Inc. (Westchester, PA) with typical transmission between 85–90%. The spacing of wires in the grids ranged from 100 lines per inch to 500 lines per inch, corresponding to a pitch of approximately 50–250 μm. The detector used to acquire images was either an x-ray CCD camera (Princeton Instruments, Model Quad-RO 4096) with a pixel size of 15 μm or an x-ray CMOS detector (Rad-Icon Imaging, RadEye200 model) with a pixel size of 98 μm. Typical exposure times were 2 h (for imaging of organs and tumors *in situ*) and 30 min (for imaging of excised organs and tumors). Long exposure times are required due to the thickness of the sample being imaged and the low power at which the x-ray tube is operated.

### Determination of gold content in tissues

The amount of gold taken up by cells or tissues after incubation or IV injection was determined by inductively-coupled plasma atomic emission spectroscopy (ICP-AES). Samples were prepared for ICP-AES analysis by digesting gold and organics with aqua regia followed by dilution in 2% nitric acid.

## Additional Information

**How to cite this article**: Rand, D. *et al.* X-ray Scatter Imaging of Hepatocellular Carcinoma in a Mouse Model Using Nanoparticle Contrast Agents. *Sci. Rep.* **5**, 15673; doi: 10.1038/srep15673 (2015).

## Figures and Tables

**Table 1:** Cellular uptake of 50 nm AuNPs with different surface chemistries by the NIH/3t3 cell line.

	Au-cit	Au-PEG-COOH
Mass of gold taken up per cell (pg)	6.9 ± 1.16	0.1 ± 0.05

**Table 2:** Changes in diameter and surface charge of 50 nm AuNPs after the addition of PEG and FB50 antibody.

	Au-cit	Au-PEG-COOH	Au-PEG-FB50
Diameter (nm)	52 ± 0.4	67 ± 0.6	76 ± 0.5
Zeta potential (mV)	−29 ± 3.3	−16 ± 1.3	−15 ± 1.9

**Table 3:** Integrated optical density measurements obtained from an immunofluorescence study of the specificity of 10 nm and 50 nm nm AuNP-antibody conjugates for HCC and control cell lines.

Nanoparticle diameter	Integrated optical density (arbitrary units)	
10 nm	Au-PEG-FB50 + FOCUS	532 ± 79.4
	Au-PEG-MUC + FOCUS	38 ± 15.0
	Au-PEG-FB50 + NIH/3t3	61 ± 33.6
50 nm	Au-PEG-FB50 + FOCUS	1460 ± 252.8
	Au-PEG-MUC + FOCUS	192 ± 101.0
	Au-PEG-FB50 + NIH/3t3	46 ± 14.1

**Table 4:** Average x-ray absorbance and x-ray scatter signal enhancements measured for organs excised from mice injected IV with 50 nm AuNPs.

	Average signal enhancement due to the presence of gold (%)		Mass of gold taken up by organ (pg)	
	X-ray absorbance	X-ray scatter	Au-PEG-COOH	Au-PEG-FB50
Liver	2.8 ± 1.26	23.0 ± 14.13	115 ± 1.1	38 ± 0.5
Kidneys	0.0 ± 7.89	6.4 ± 11.91	3 ± 0.1	3 ± 0.1
Spleen	4.3 ± 8.34	5.1 ± 29.81	30 ± 0.1	13 ± 0.1
Lungs	1.1 ± 9.09	0.9 ± 31.52	1 ± 0.1	0 ± 0.1

**Table 5:** Masses of gold delivered to tumor site and liver *in vivo.*

	Au-PEG-COOH tumor	Au-PEG-FB50 tumor 1	Au-PEG-FB50 tumor 2
Mass of Au per volume tissue (μg/cm^3^)	18 ± 7.0	26 ± 3.3	24 ± 1.0
Ratio to Au-PEG tumor	1.00	1.45	1.33
Mass of Au per volume tissue in corresponding liver (μg/cm^3^)	25 ± 0.2	23 ± 0.6	23 ± 0.6
Ratio of tumor to corresponding liver	0.71	1.12	1.02
