# Corrigendum: X-ray Scatter Imaging of Hepatocellular Carcinoma in a Mouse Model Using Nanoparticle Contrast Agents

**DOI:** 10.1038/srep21229

**Published:** 2016-03-10

**Authors:** Danielle Rand, Zoltan Derdak, Rolf Carlson, Jack R. Wands, Christoph Rose-Petruck

Scientific Reports 5: Article number: 15673; 10.1038/srep15673 published online 10292015; updated: 03102016

The Acknowledgements section is incomplete.

“C.R.P. acknowledges financial support for the development of the X-ray imaging modality by the U.S. Department of Energy under Grant DE-FG02-08ER15937. J.R.W. acknowledges financial support by the National Institutes of Health under Grant CA123544. The authors thank Virginia Hovanesian for her assistance with immunofluorescence studies.”

should read:

“C.R.P. acknowledges financial support for the development of the X-ray imaging modality by the U.S. Department of Energy under Grant DE-FG02-08ER15937. J.R.W. acknowledges financial support by the National Institutes of Health under Grant CA123544. The authors thank Virginia Hovanesian for her assistance with immunofluorescence studies. The authors also thank Vivian Ortiz for her help with data collection and figure preparation, and for valuable discussions on these topics.”

